# Adverse effects of antipsychotics on sleep in patients with schizophrenia. Systematic review and meta-analysis

**DOI:** 10.3389/fpsyt.2023.1189768

**Published:** 2023-06-27

**Authors:** Yarmila Elena Valencia Carlo, Ricardo Arturo Saracco-Alvarez, Verónica Angela Valencia Carlo, Daniela Vázquez Vega, Guillermina Natera Rey, Raul Ivan Escamilla Orozco

**Affiliations:** ^1^Health Sciences Program, Universidad Nacional Autonoma de Mexico, Mexico City, Mexico; ^2^Clinical Research, National Institute of Psychiatry Ramon de la Fuente Muñiz, Mexico City, Mexico; ^3^Hematology Services, Clinical Hospital, La Paz, Bolivia; ^4^Department of Epidemiological and Psychosocial Research, National Institute of Psychiatry Ramon de la Fuente Muñiz, Mexico City, Mexico; ^5^Ambulatory Services, National Institute of Psychiatry Ramon de la Fuente Muñiz, Mexico City, Mexico

**Keywords:** antipsychotics, schizophrenia, insomnia, somnolence, sedation

## Abstract

**Introduction:**

Our objective was to conduct a systematic review and meta-analysis of adverse effects on sleep in patients with schizophrenia receiving antipsychotic treatment.

**Methods:**

A systematic search was performed in PubMed, Cochrane Central, Embase, Toxline, Ebsco, Virtual Health Library, Web of Science, SpringerLink, and in Database of abstracts of Reviews of Effects of Randomized Clinical Trials to identify eligible studies published from January 1990 to October 2021. The methodological quality of the studies was evaluated using the CONSORT list, and the Cochrane bias tool. Network meta-analysis was performed using the Bayesian random-effects model, with multivariate meta-regression to assess the association of interest.

**Results:**

87 randomized clinical trials were identified that met the inclusion criteria, and 70 articles were included in the network meta-analysis. Regarding the methodological quality of the studies, 47 had a low or moderate bias risk. The most common adverse effects on sleep reported in the studies were insomnia, somnolence, and sedation. The results of the network meta-analysis showed that ziprasidone was associated with an increased risk of insomnia (OR, 1.56; 95% credible interval CrI, 1.18–2.06). Several of the included antipsychotics were associated with a significantly increased risk of somnolence; haloperidol (OR, 1.90; 95% CrI, 1.12–3.22), lurasidone (OR, 2.25; 95% CrI, 1.28–3.97) and ziprasidone (OR, 1.79; 95% CrI, 1.06–3.02) had the narrowest confidence intervals. In addition, perphenazine (OR, 5.33; 95% CrI, 1.92–14.83), haloperidol (OR, 2.61; 95% CrI, 1.14–5.99), and risperidone (OR, 2.41; 95% CrI, 1.21–4.80) were associated with an increased risk of sedation compared with placebo, and other antipsychotics did not differ. According to the SUCRAs for insomnia, chlorpromazine was ranked as the lowest risk of insomnia (57%), followed by clozapine (20%), while flupentixol (26 %) and perospirone (22.5%) were associated with a lower risk of somnolence. On the other hand, amisulpride (89.9%) was the safest option to reduce the risk of sedation.

**Discussion:**

Insomnia, sedation, and somnolence were the most frequent adverse effects on sleep among the different antipsychotics administered. The evidence shows that chlorpromazine, clozapine, flupentixol, perospirone, and amisulpride had favorable safety profiles. In contrast, ziprasidone, perphenazine, haloperidol, and risperidone were the least safe for sleep.

**Systematic review registration:**

https://www.crd.york.ac.uk/prospero/display_record.php?ID=CRD42017078052, identifier: PROSPERO 2017 CRD42017078052.

## Introduction

Schizophrenia is a psychiatric disorder that affects ~1% of the world population and is characterized by positive or psychotic symptoms (e.g., hallucinations, delusions, and disorganized thoughts and/or disorganized speech), negative symptoms (e.g., decreased motivation, decreased emotional expressiveness, autism and social withdrawal), and cognitive deficits (e.g., impaired executive functions, memory, and processing speed) (1).

Patients with schizophrenia frequently report sleep problems. There are numerous factors contributing to the comorbidity, including medication intake (2). Although AP medications have prominent effect of sleep, however, have also demonstrated beneficial by increasing sleep efficiency and total sleep time, and reducing sleep onset latency (SOL) (3, 4).

Antipsychotics (AP) are essential component of schizophrenia treatment. These drugs are classified into first-generation antipsychotics (FGAs) and second-generation antipsychotics (SGAs), according to their molecular mechanism of action. As with other medications, AP despite their proven therapeutic utility, can also generate multiple adverse drug reactions (ADRs), ranging from metabolic to motor effects (5). Many of these consequences affect the patients' lifestyle, contribute to the rate of abandonment of treatment, and influence the prognosis of the disease (6). Some studies focusing on antipsychotic medication have demonstrated that adverse effects have a negative impact on patient adherence to treatment, both in institutional and outpatient setting. Thus, it is in the best interest of the patient and of the clinical staff to reduce adverse effects and prevent their recurrence (7, 8).

The results of some studies showed that increasing antipsychotic dosages were linked to better sleep, although antipsychotic medications only accounted for a relatively small amount of the variance in sleep (8). Furthermore, sleep complaints were pervasive in 70% of this medicated clinical sample. These findings suggest that the use of the sedative properties of antipsychotic medication has limited efficacy as a treatment option for sleep dysfunctions, and is not an appropriate substitute for sleep interventions (9, 10). The degree of discomfort caused by each type of side effect is variable in patient reports; (11) certain ones report indicate that most patients find extrapyramidal effects, sexual dysfunction, and weight gain to be the most intolerable, as opposed to sedation and other vegetative effects. The presence of these effects is associated with poor treatment adherence (12), and some of these effects may be therapeutic for people with schizophrenia, such as sedation in those with insomnia or perhaps the inversion of sleep architecture seen in some patients, since the pharmacological effects of medications used to treat schizophrenia can affect sleep-wake function. In general, FGAs are linked to increased REM sleep latency and SGAs to increased slow-wave sleep reduction (13, 14).

Another possible AP adverse effect is somnolence. It can be defined as a propensity to fall asleep and while in this state, conscious effort may need to be made to stay awake; in contrast to fatigue which is more related to physical exhaustion (15). All antipsychotics have been observed to cause sedation, but the severity and frequency varies widely between agents (16). Although it is a common adverse effect and a common reason for medication non-adherence, the evidence for sedation has not been widely examined in systematic reviews. Sedation, instead, is a term denoting the use of pharmacological agents to calm acutely agitated patients, both in a psychiatric care service as well as in general acute care setting (17). Somnolence and sedation are related to the delivered dose and are the most bothersome problems for patients, compared to insomnia, which is also common with AP medications, and may be the cause of poor adherence to treatment, if persistent, may interfere with the individual's social and vocational functioning (18). A systematic review with meta-analysis by Andrade et al. (19) found no significant differences in daytime sleepiness between placebo and antipychotics associated with modafinil or armodafinil using a random effects model (mean difference = 0.78; 95% CI = 3.33–1.76).

Antipsychotic medication has an effect on various neurotransmitter receptors such as acetylcholine, dopamine, histamine, norepinephrine, and serotonin. For example, serotonin receptor antagonism promotes sedation and may increase slow-wave sleep, whereas 5-HT1A receptors agonism may cause sleep disturbances and suppress REM sleep. Blockage of histamine receptors promotes sedative and sleep-enhancing effects, can reduce REM sleep intensity, and increase REM latency (20). Antagonism of dopamine receptors can result in restless leg syndrome, which may interfere with sleep (21). Aside from antidopaminergic activity, many other receptors bind to almost all dopamine receptor antagonists or APs, including cholinergic receptors (22). There is an inverse relationship between the potency of the antipsychotic and blockade of these receptors, so those with less D2 affinity are the ones that most frequently generate somnolence. For its management, it is recommended to administer the entire dose or most of it at night. If somnolence is incapacitating, it is recommended to reduce the dose of the DRA or change it to another with less drowsiness effect (23, 24).

According to polysomnographic studies, administration of some AP such as clozapine, paliperidone and olanzapine to patients with schizophrenia frequently results in a significant reduction of sleep latency (SL) and increase in total sleep time and sleep efficiency. In addition, olanzapine and paliperidone augmented slow wave sleep (SWS) and REM sleep. In contrast, quetiapine administration further disrupted sleep measured by reduction of SWS and REM sleep, also for the increase of SL, wake time after onset (WASO) and REM sleep latency (25, 26).

The variability in the AP effects on sleep structure appears to be related to the individual receptor binding profiles of the drugs for the mentioned neurotransmitters (26). Indeed, clinicians acknowledge the prevalence and importance of sleep problems in patients, but in current practice formal assessment is rare and evidence is limited. However, there is clear interest in treating this side effect, as three quarters of patients with psychosis who have insomnia would like to improve their sleep (27).

Reviewing the studies published in the last 20 years, we tried to answer the following question: what are the adverse effects of typical and atypical antipsychotics on the sleep of patients diagnosed with schizophrenia? The aim of this research, therefor, was to perform a systematic review and meta-analysis of adverse effects on sleep in patients with schizophrenia receiving antipsychotic treatment.

## Materials and methods

The research protocol was registered on the PROSPERO platform (CRD42017078052), developed by the University of York (http://www.crd.york.ac.uk/prospero/). A search was made for studies published between 1990 and October 2021, using the following electronic databases: Pubmed, Embase, Cochrane library, Ebsco, Toxline, Springerlink, Web of Science, Database of abstracts of Reviews of Effects and the Virtual Health Library; Abstracts.

The search strategy was carried out with the following terms: (*schizophrenia AND antipsychotic agents AND sleep disorders OR sleep disturbances OR sleep effects OR sleep wake up disorders*) and (*Schizophrenia AND antipsychotic effects AND Sleep*), and in Spanish (*Esquizofrenia AND Agentes Antipsicóticos AND Trastornos del Sueño OR problemas de sueño OR efectos en el sueño OR trastornos del sueño vigilia*), and (*Esquizofrenia AND efectos de antipsicóticos AND sueño*).

### Inclusion and exclusion criteria

The inclusion criteria of our systematic review were: (a) randomized and controlled clinical trials in which antipsychotic treatment is provided, in monotherapy or together with an adjuvant medication, (b) written in English or Spanish, (c) studies in which to people ≥18 years, diagnosed with schizophrenia, regardless of subtype or severity of symptoms, (d) trials that reported adverse effects on sleep related to the use or withdrawal of any antipsychotic. As exclusion criteria we have (a) observational studies, case reports, systematic reviews or meta-analysis, (b) investigations with incomplete or unclear methodology, (c) those that do not analyze the relationship between antipsychotics and adverse effects in sleep, and (d) inclusion of participants with other psychiatric disorders, medical or neurological conditions.

### Information extraction

**Figure 1:**
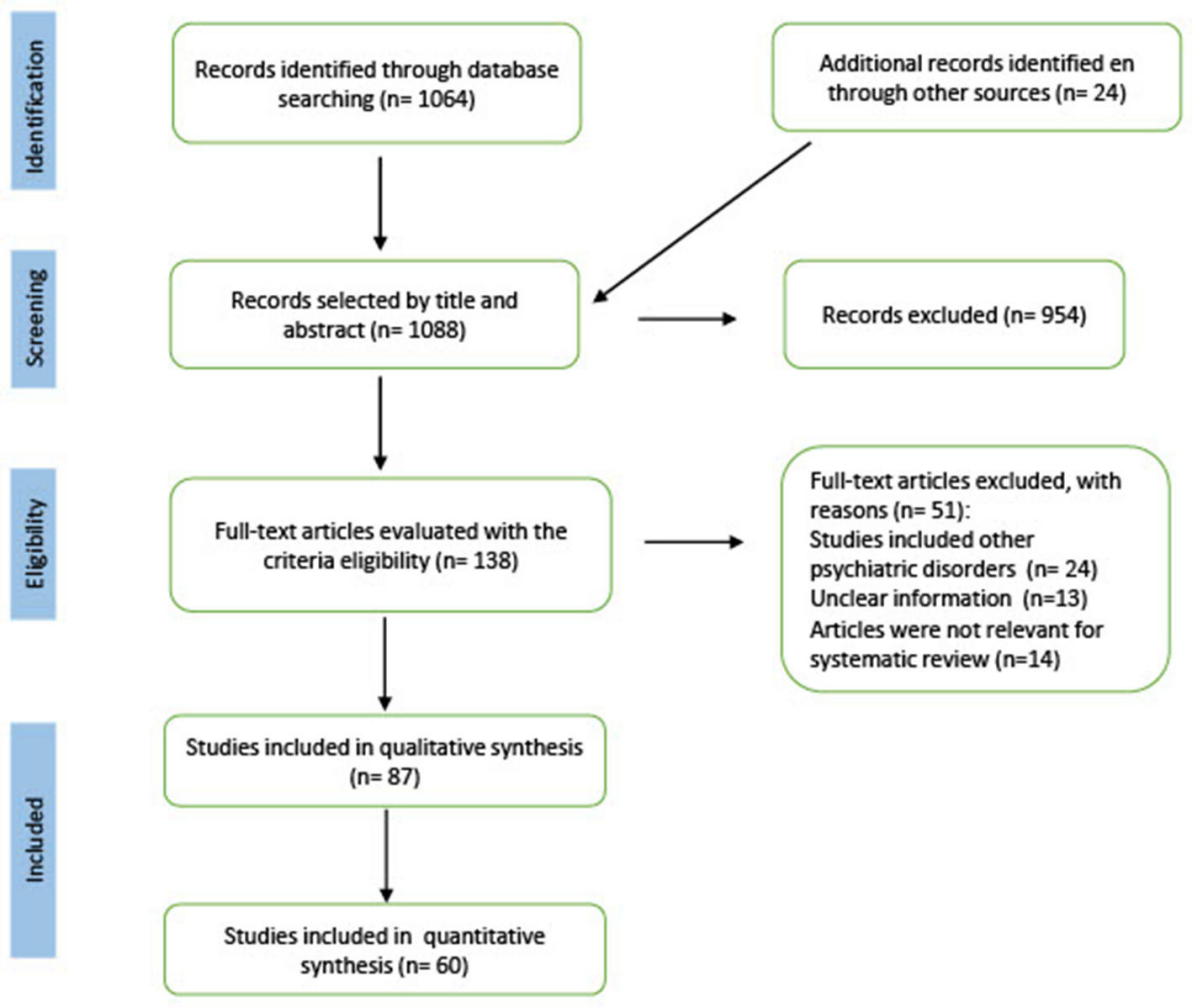
PRISMA flow diagram of study selection. A PRISMA flowchart showing the number of articles selected in each phase, the number of articles excluded, and the details of all studies included and excluded from the systematic review.

Initially, titles and abstracts were initially reviewed, in a second step, the extensive content of the potentially eligible articles was evaluated, in pairs and independently. In the event of discrepancies regarding the inclusion of a study in the review, another of the authors served as the third evaluator to reach consensus. Using the bibliographical references of these articles, we searched twenty-four additional studies to verify if they met inclusion criteria. For that, a flow chart (PRISMA) of the number of articles selected in each phase, of those eliminated and the reason for exclusion was developed. Figure 1 shows the details of all the studies included in the systematic review.

To carry out the qualitative synthesis of the results, the following data were extracted in a table of variables: authors, year of publication, country of origin, prescribed antipsychotics, sample size, comparison groups, frequency of adverse effects on sleep, loss to follow-up, funding, conflict of interest and protocol registration number (see Data extraction in the Supplementary material).

### Methodological quality

We evaluated the quality of the publications through the checklist of the CONSORT statement (28). For each item, three types of response were recorded: “yes”, “does not include” and “not applicable”, following the recommendations on how to evaluate the CONSORT items of the article published by Moher et al. (29). Some items on the checklist have multiple components, so if at least half of them were met, we scored that item “yes.” If less than half of the components were fulfilled or a key component was missing or the information was unclear, the item was scored as “does not include”, then the scores of all the items were added to obtain an overall score and this was expressed in percentage.

Three quality assessment categories were used that were applied in other published research: (1) the study meets more than 80% of the criteria, (2) the study meets between 50 and 80% of the criteria, and (3) the study meets <50% of the criteria identified in the CONSORT checklist (30). To assess the individual risk of bias for each clinical trial, we applied the Cochrane collaboration risk of bias tool. This tool poses six levels of bias: selection, performance, detection, attrition, reporting, and other biases. The response options of the level of bias were classified as: (1) high risk of bias, in this case the bias seriously weakens the confidence in the results, (2) unclear risk, the bias causes some doubt about the results and (3) low risk, refers to the fact that the bias is unlikely to significantly alter the results (28, 30).

### Network meta-analysis

The network meta-analysis (NMA) generates evidence from direct and indirect comparisons within a network of trials and enables inference about every possible comparison between a pair of interventions in this network, even when some comparisons have never been directly evaluated in a trial (31).

The NMA resulted in a complex network, in which direct comparisons for each antipsychotic are observed when comparing them individually against placebo. The use of placebo in RCT is widely considered the gold standard for testing the efficacy of new treatments, beyond the psychological results of a simple belief in the curative capacity of the drug, the researchers compare the results of the experimental treatment with those obtained with the placebo (32, 33). It is also useful when the treatment used is of questionable efficacy or has a high frequency of adverse reactions and the risks may significantly outweigh the benefits (34).

To present the evidence graphically, we used network plots of antipsychotic interventions, which provide a visual representation of the evidence base and a description of its characteristics. A network plot consists of nodes representing the interventions being compared, and edges representing the available direct comparisons between pairs of interventions (35). Each treatment node or comparison edge can be weighted according to the number of trials and the average control group risk for placebo vs. active treatment comparisons (36). We estimated summary odds ratios (ORs) for dichotomous outcomes with a 95% credible interval.

We estimated summary odds ratios (ORs) for dichotomous outcomes with a 95% credible interval.

Consistency was evaluated by examining the agreement between direct and indirect treatment effects in all closed loops and assuming loop-specific heterogeneity using the loop-specific approach. To evaluate the presence of inconsistency for any treatment contrast in the network, the node-splitting analysis method was applied.

In addition to OR estimation, treatment ranking was ascertained using the surface under the cumulative ranking curve (SUCRA), which represents the percentage of effectiveness or safety for each treatment compared with a hypothetical treatment that would be ranked first without uncertainty (37). We also plot rankograms, which are graphs that show the probability that each treatment has of reaching a certain ranking. On the *X*-axis they show the possible ranking and on the *Y*-axis the probability that each treatment has of reaching it (38).

### Statistical analysis

A Bayesian network meta-analysis based on the random effects model was performed using the STATA 16.1 program. In this analysis the treatment arms with the maximum dose of each antipsychotic and placebo were included. Effect sizes and 95% credible intervals were calculated for the selected articles. The ranking of the different treatments was based on the calculation of the areas under the cumulative rank probability curve or surface under the cumulative ranking curve (SUCRA) (39), which is expressed as a percentage and is interpreted as the probability that all other treatments are inferior or the probability (Pr) of being the best treatment (38).

## Results

### Characteristics of the studies

Of the 1,088 studies initially identified in the search, we excluded 954 studies after reviewing the title, abstract and keywords, as they did not meet the inclusion criteria. During the manual search we found eight eligible studies, of which two were included in the systematic review.

Our review found that 87 clinical trials (40–126) met the established inclusion criteria, 3,813 participants (13.82%) had adverse effects on sleep out of a total of 27,595 people who received treatment with any antipsychotic medication (Table 1). Insomnia (85.54%), somnolence (43.37%) and sedation (16.87%) were the most common adverse events (see Table 2). Other unspecified sleep problems were reported in the articles, but to a lesser extent (3.45%).

**Table 1:** Articles included in the systematic review (*n* = 87) (40–126).

**References**	***n***	**Adverse effects on sleep** ***n*** **(%)**			**Dose**
Abdolahian et al. (40)	65	**Insomnia**			
		Haloperidol: 17/30 (56.67)			Haloperidol: 10–15 mg/d
		Risperidone: 13/35 (37.14)			Risperidone: 4–8 mg/d
Álvarez et al. (41)	170	**Insomnia**			
		Olanzapine: 17/124 (13.71)			Olanzapine: 5–30 mg/d
		Risperidone: 8/123 (6.50)			Risperidona: 2–9 mg/d
Arato et al. (42)	278	**Insomnia**			
		Ziprasidone 40 mg: 20/72 (27.78)			Ziprasidone: 40 mg, 80 mg, or 160 mg/d
		Ziprasidone 80 mg: 19/68 (27.94)			
		Ziprasidone 160 mg: 30/67 (44.77)			
		Placebo: 22/71 (30.99)			Placebo
Azorin et al. (43)	186	**Insomnia**	**Somnolence**	**Other sleep disorder**	
		Risperidone: 11/89 (12.36)	Risperidone: 7/89 (7.86)	Risperidone: 5/89 (5.62)	Risperidone: 4–10 mg/d
		Sertindole: 9/97 (9.27)	Sertindole: 8/97 (8.24)	Sertindole: 2/97 (2.06)	Sertindole: 12–24 mg/d
Beasley et al. (44)	335	**Insomnia**	**Somnolence**		
		Haloperidol: 14/69 (20.29)	Haloperidol: 24/69 (34.78)		Haloperidol: 15 mg ± 5 mg/d
		Olanzapine 5 mg: 14/65 (21.54)	Olanzapine 5 mg: 13/65 (20)		Olanzapine: 5 ± 2.5 mg, 10 ± 2.5 mg, or 15 ± 2.5 mg
		Olanzapine 10 mg: 16/64 (25)	Olanzapine 10 mg: 27/64 (42.18)		
		Olanzapine 15 mg: 12/69 (17.39)	Olanzapine 15 mg: 27/69 (39.13)		
		Placebo: 15/68 (22.06)	Placebo: 11/68 (16.17)		Placebo: Equivalent dose
Berwaerts et al. (45)	305	**Insomnia**			
		Paliperidone: 11/160 (6.87)^*^			Paliperidone: 175–525 mg eq/month
		Placebo: 17/145 (11.72)^*^			Placebo: Intralipid 20%
Bitter et al. (46)	150	**Somnolence**			
		Olanzapine: 2/76 (2.63)			Olanzapine: 5–25 mg/d
		Clozapine: 11/74 (14.86)			Clozapine: 100–500 mg/d
Breier et al. (47)	548	**Insomnia**			
		Olanzapine: 19/277 (6.86)			Olanzapine: 10–20 mg/d
		Ziprasidone: 60/271 (22.14)			Ziprasidone: 80–160 mg/d
Bhowmick et al. (48)	80	**Insomnia**	**Sedation**		
		Amisulpride: 7/40 (17.5)	Amisulpride: 0/40 (0)		Amisulpride: 100–800 mg/d
		Olanzapine: 1/40 (2.50)	Olanzapine: 10/40 (25.0)		Olanzapine: 10–20 mg/d
Chue et al. (49)	640	**Insomnia**			
		Risperidone: 29/321 (9.03)			Risperidone: 1–6 mg/d
		Risperidone LAI: 31/319 (9.72)^*^			Risperidone LAI: 25–75 mg/2 weeks
Colonna et al. (50)	488	**Insomnia**			
		Haloperidol: 15/118 (12.71)			Haloperidol: 5–20 mg/d
		Amisulpride: 50/370 (13.51)			Amisulpride: 200–800 mg/d
Cooper et al. (51)	119	**Insomnia**	**Somnolence**		
		Zotepine: 7/61 (11.47)	Zotepine: 31/61 (50.82)		Zotepine: 150–300 mg/d
		Placebo: 8/58 (13.79)	Placebo: 10/58 (17.24)		Placebo: Equivalent dose
Conley et al. (52)	84	**Insomnia**	**Somnolence**		
		Olanzapine: 6/42 (14.29)	Olanzapine: 15/42 (35.71)		Olanzapine: 12.5–25 mg/d
		Chlorpromazine: 2/42 (4.76)	Chlorpromazine: 22/42 (52.38)		Chlorpromazine: 600–1200 mg/d
Correll et al. (53)	636	***Insomnia***	**Somnolence**	**Sedation**	
		Brexpiprazole 0.25: 8/90 (8.89)	Brexpiprazole 0.25 mg: 0/90 (0)	Brexpiprazole: 4/180 (2.22)	Brexpiprazole: 0.25 mg, 2 mg, or 4 mg/d
		Brexpiprazole 2 mg: 16/182 (8.79)	Brexpiprazole 2 mg: 3/182 (1.65)		
		Brexpiprazole 4 mg: 15/180 (8.33)	Brexpiprazole 4 mg: 7/180 (3.89)		
		Placebo: 18/184 (9.78)	Placebo: 5/184 (2.72)	Placebo: 1/184 (0.54)	Placebo
Cutler et al. (54)	365	**Sedation**			
		Aripiprazole 2 mg: 4/93 (4.30)			Aripiprazole: 2 mg, 5 mg, or 10 mg/d
		Aripiprazole 5 mg: 6/91 (6.59)			
		Aripiprazole 10 mg: 3/94 (3.19)			
		Placebo: 0/87 (0)			Placebo: Equivalent dose
Cutler et al. (55)	597	**Somnolence**	**Sedation**		
		Iloperidone: 12/300 (4.0)	Iloperidone: 38/300 (12.67)		Iloperidone: Hasta 24 mg/d
		Ziprasidone: 9/150 (6.0)	Ziprasidone: 41/150 (27.33)		Ziprasidone: Hasta 160 mg/d
		Placebo: 2/147 (1.36)	Placebo: 12/147 (8.16)		Placebo: Equivalent dose
Davidson et al. (56)	614	**Insomnia**	**Somnolence**		
		Olanzapine: 16/127 (12.59)	Olanzapine: 22/127 (17.32)		Olanzapine: 10 mg/d
		Paliperidone ER 3 mg: 15/127 (11.81)	Paliperidone ER 3 mg: 7/127 (5.51)		Paliperidone ER: 3 mg, 9 mg, or 15 mg/d
		Paliperidone ER 9 mg: 18/124 (14.51)	Paliperidone ER 9 mg: 16/124 (12.90)		
		Paliperidone ER 15 mg: 18/113 (15.93)	Paliperidone ER: 10/113 (8.85)		
		Placebo: 17/123 (13.82)	Placebo: 4/123 (3.25)		Placebo: Equivalent dose
Detke et al. (57)	524	**Insomnia**	**Somnolence**		
		Olanzapine: 26/260 (10.0)	Olanzapine: 25/260 (9.61)		Olanzapine: 5–20 mg/d
		Olanzapine LAI: 29/264 (10.98)^*^	Olanzapine LAI: 23/264 (8.71)^*^		Olanzapine LAI: 150–405 mg/month
Dossenbach et al. (58)	60	**Insomnia**			
		Olanzapine: 0/30 (0)			Olanzapine: 5–20 mg/d
		Fluphenazine: 6/30 (20)			Fluphenazine: 6–21 mg/d
Downing et al. (59)	437	**Insomnia**			
		Risperidone: 10/142 (7.04)			Risperidone: 2 mg/BID
		Placebo: 21/295 (7.12)			Placebo: Equivalent dose
Durgam et al. (60)	729	**Insomnia**	**Sedation**		
		Risperidone: 21/140 (15.0)	Risperidone: 16/140 (11.43)		Risperidone: 4 mg/d
		Cariprazine 1.5 mg: 15/145 (10.34)	Cariprazine 1.5 mg: 7/145 (4.83)		Cariprazine: 1.5 mg, 3 mg, or 4.5 mg/d
		Cariprazine 3 mg: 24/146 (16.44)	Cariprazine 3 mg: 7/146 (4.79)		
		Cariprazine 4.5 mg: 24/147 (16.32)	Cariprazine: 12/147 (8.16)		
		Placebo: 11/151 (7.28)	Placebo: 5/151 (3.31)		Placebo: Equivalent dose
Durgam et al. (61)	617	**Insomnia**			
		Aripiprazole: 16/152 (10.52)			Aripiprazole: 10 mg/d
		Cariprazine 3 mg: 21/155 (13.55)			Cariprazine: 3 mg, or 6 mg/d
		Cariprazine 6 mg: 22/157 (14.01)			
		Placebo: 25/153 (16.34)			Placebo: Equivalent dose
Durgam et al. (62)	389	**Insomnia**			
		Cariprazine 1.5–4.5 mg: 12/127 (9.45)			Cariprazine: 1.5–4.5 mg/d or 6–12 mg/d
		Cariprazine 6–12 mg: 16/133 (12.03)			
		Placebo: 10/129 (7.75)			Placebo
Durgam et al. (63)	200	**Insomnia**			
		Cariprazine: 8/101 (7.92)			Cariprazine: 3–9 mg/d
		Placebo: 8/99 (8.08)			Placebo: Equivalent dose
Emsley et al. (64)	288	**Insomnia**	**Somnolence**		Haloperidol: 20 mg/d
		Haloperidol: 9/145 (6.21)	Quetiapine: 14/143 (9.79)		Quetiapine: 600 mg/d
Fleischhacker et al. (65)	747	**Insomnia**			
		Paliperidone: 57/379 (15.04)^*^			Paliperidone: 25–100 mg eq./month
		Risperidone LAI: 55/368 (14.94)^*^			Risperidone LAI: 25–50 mg/2 weeks
Fleischhacker et al. (66)	662	**Insomnia**			
		Aripiprazole: 37/266 (13.91)			Aripiprazole: 10–30 mg/d
		Aripiprazole LAI 50 mg: 18/131 (13.74)			Aripiprazole LAI: 50 mg/month or 400 mg/month
		Aripiprazole LAI 400 mg: 31/265 (11.69)			
Garcia et al. (67)	188	**Insomnia**	**Somnolence**		
		Blonanserin: 6/64 (9.38)	Blonanserin: 4/64 (6.25)		Blonanserin: 10 mg/d
		Haloperidol: 3/60 (5.0)	Haloperidol: 2/60 (3.33)		Haloperidol: 10 mg/d
		Placebo: 6/64 (9.38)	Placebo: 2/64 (3.12)		Placebo: Equivalent dose
Gattaz et al. (68)	28	**Somnolence**			
		Olanzapine: 2/15 (13.33)			Olanzapine: 5–20 mg/d
		Flupentixol: 1/13 (7.69)			Flupentixol: 5–20 mg/d
Golden (69)	70	**Somnolence**			
		Clozapine: 7/36 (19.44)			Clozapine: 100 mg/d BID
		Clozapine dispersible: 5/3 (14.71)			Clozapine dispersible: 100 mg/d BID
Hirsch et al. (70)	301	**Insomnia**	**Somnolence**		
		Ziprasidone: 24/148 (16.22)	Ziprasidone: 20/148 (13.51)		Ziprasidone: 80–160 mg/d
		Haloperidol: 27/153 (17.65)	Haloperidol: 13/153 (8.49)		Haloperidol: 5–15 mg/d
Hough et al. (71)	252	**Insomnia (1st application)**			
		Paliperidone palmitate^*^			Paliperidone: 50, 75 and 100 mg eq deltoid/gluteus (DG)
		Deltoid muscle: 18/128 (14.06)			
		Gluteus muscle: 19/124 (15.32)			
		**Insomnia (2nd application)**			
		Paliperidone palmitate^*^			Paliperidone: 50, 75 y 100 mg eq gluteus/deltoid (GD)
		Gluteus muscle: 6/128 (4.69)			
		Deltoid muscle: 10/124 (0.80)			
Huang et al. (72)	57	**Insomnia**	**Somnolence**		
		Paliperidone LAI: 6/28 (21.43)^*^	Paliperidone LAI: 4/28 (14.28)^*^		Paliperidone LAI: Mean dose 128.85 (± 28.01) mg eq/month
		Olanzapine: 17/29 (58.62)	Olanzapine: 1/29 (3.45)		Olanzapine: Mean dose 17.80 (± 3.56) mg/d
Hwang et al. (73)	70	**Insomnia**	**Somnolence**		
		Zotepine: 3/35 (.8.57)	Zotepine: 11/35 (31.43)		Zotepine: 150 mg/d
		Haloperidol: 4/35 (11.43)	Haloperidol: 7/35 (20.0)		Haloperidol: 9 mg/d
Ishigooka et al. (74)	455	**Insomnia**			
		Aripiprazole: 20/227 (8.81)			Aripiprazole: 6–24 mg/d
		Aripiprazole LAI: 17/228 (7.45)^*^			Aripiprazole LAI: 400 mg/month
Kahn et al. (75)	588	**Insomnia**	**Somnolence**	**Other sleep disorder**	
		Quetiapine IR: 13/123 (10.57)	Quetiapine IR: 9/123 (7.32)	Quetiapine IR: 6/123 (4.87)	Quetiapine IR: 400 mg/d
		Quetiapine XR 400 mg: 13/113 (11.50)	Quetiapine XR 400 mg: 8/113 (7.08)	Quetiapine XR 400 mg: 4/113 (3.54)	Quetiapine XR: 400 mg, 600 mg, or 800 mg/d
		Quetiapine XR 600 mg: 7/113 (6.19)	Quetiapine XR 600 mg: 10/113 (8.85)	Quetiapine XR 600 mg: 6/113 (5.31)	Placebo: Equivalent dose
		Quetiapine XR 800 mg: 9/121 (7.43)	Quetiapine XR 800 mg: 14/121 (11.57)	Quetiapine XR 800 mg: 4/121 (3.31)	
		Placebo: 23/118 (19.49)	Placebo: 2/118 (1.69)	Placebo: 2/118 (1.69)	
Kane et al. (76)	400	**Insomnia**	**Somnolence**		
		Risperidone LAI 25 mg: 16/99 (16.16)^*^	Risperidone LAI 25 mg: 5/99 (5.05)^*^		Risperidone LAI: 25 mg, 50 mg, or 75 mg/2 weeks
		Risperidone LAI 50 mg: 13/103 (12.62)^*^	Risperidone LAI 50 mg: 6/103 (5.83)^*^		
		Risperidone LAI 75 mg: 16/100 (16.0)^*^	Risperidone LAI 75 mg: 10/100 (10)^*^		
		Placebo: 14/98 (14.28)^*^	Placebo: 3/98 (3.06)^*^		Placebo: Equivalent dose
Kane et al. (77)	629	**Insomnia**	**Somnolence**		
		Olanzapine: 18/128 (14.06)	Olanzapine: 18/128 (14.06)		Olanzapine: 10 mg/d
		Paliperidone: 16/130 (12.31)	Paliperidone: 10/130 (7.69)		Paliperidone ER: 6 mg, 9 mg, and 12 mg/d
		Placebo: 22/126 (17.46)	Placebo: 7/126 (5.56)		Placebo: Equivalent dose
Kane et al. (78)	921	**Insomnia**	**Somnolence**		
		Olanzapine tab: 13/322 (4.03)	Olanzapine tab: 9/322 (2.79)		Olanzapine tab: 10–20 mg/d
		Olanzapine LD IM: 11/140 (7.85)^*^	Olanzapine LD IM: 8/140 (5.71)^*^		Olanzapine LD.: 150 mg/2 weeks
		Olanzapine MD IM: 23/318 (7.23)^*^	Olanzapine MD IM: 10/318 (3.14)^*^		Olanzapine MD: 405 mg/4 weeks
		Olanzapine HD IM: 9/141 (6.38)^*^	Olanzapine HD IM: 5/141 (3.54)^*^		Olanzapine HD: 300 mg/2 weeks
Kane et al. (79)	386	**Insomnia**			
		Asenapine: 1/194 (0.51)			Asenapine: 20 mg/d
		Placebo: 2/192 (1.04)			Placebo: Equivalent dose
Kane et al. (80)	403	**Insomnia**			
		Aripiprazole LAI: 27/269 (10.03)^*^			Aripiprazole: 300–400 mg/month
		Placebo: 12/134 (8.96)^*^			Placebo: Equivalent dose
Kane et al. (81)	674	**Insomnia**	**Somnolence**	**Sedation**	
		Brexpiprazole 1 mg: 15/120 (12.5)	Brexpiprazole 1 mg: 2/120 (1.67)	Brexpiprazole 1 mg: 2/120 (1.67)	Brexpiprazole: 1 mg, 2 mg, or 4 mg
		Brexpiprazole 2 mg: 25/186 (13.44)	Brexpiprazole 2 mg: 3/186 (1.61)	Brexpiprazol 2 mg: 6/186 (3.23)	
		Brexpiprazole 4 mg: 28/184 (15.22)	Brexpiprazole 4 mg: 5/184 (2.71)	Brexpiprazole 4 mg: 6/184 (3.26)	
		Placebo: 27/184 (14.67)	Placebo: 27/184	Placebo: 2/184 (1.08)	Placebo: Equivalent dose
Kramer et al. (82)	206	**Insomnia**			
		Paliperidone: 5/104 (4.81)			Paliperidone ER: 3–15 mg/d
		Placebo: 6/102 (5.88)			Placebo: Equivalent dose
Kasper et al. (83)	1,290	**Insomnia**	**Somnolence**		
		Haloperidol: 88/431 (20.42)	Haloperidol: 32/431 (7.42)		Haloperidol: 10 mg/d
		Aripiprazole: 185/859 (21.53)	Aripiprazole: 43/859 (5.0)		Aripiprazole: 30 mg/d
Kerwin et al. (84)	548	**Insomnia**	**Somnolence**		
		Aripiprazole: 68/282 (24.11)	Aripiprazole: 11/282 (3.90)		Aripiprazole: 15–30 mg/d
		Other antipsychotics: 20/266 (7.52)	Other antipsychotics: 31/266 (11.65)		Other antipsychotics: Olanzapine 5–20 mg/d, quetiapine 100–800 mg/d, and risperidone 2–16 mg/d
Lal et al. (85)	31	**Insomnia**			
		Chlorpromazine: 8/14 (57.14)			Chlorpromazine: Mean dose 722 mg/d (SD: 265)
		Levomepromazine: 8/17 (47.05)			Levomepromazine: Mean dose 710 mg/d (SD: 272)
Landbloom et al. (86)	357	**Somnolence**			
		Olanzapine: 5/46 (10.87)			Olanzapine: 15 mg/d
		Asenapine 2.5 mg: 3/97 (3.09)			Asenapine: 2.5 mg/d BID
		Asenapine 5 mg: 7/113 (6.19)			Asenapine: 5 mg/d BID
		Placebo: 0/101 (0)			Placebo: Equivalent dose
Lecrubier et al. (87)	245	**Insomnia**			
		Amisulpride: 14/70 (20.0)			Amisulpride: 150 mg/d
		Olanzapine 5 mg: 13/70 (18.57)			Olanzapine: 5 mg or 20 mg/d
		Olanzapine 20 mg: 4/70 (5.71)			
		Placebo: 9/35 (25.71)			Placebo
Li et al. (88)	452	**Insomnia**			
		Paliperidone ER: 11/229 (4.80)^*^			Paliperidone ER: 50–150 mg eq/month
		Risperidone LAI: 6/223 (2.69)^*^			Risperidone LAI: 25–50 mg/2 weeks
Li et al. (89)	119	**Insomnia**			
		Risperidone: 3/59 (5.08)			Risperidone: 3–4 mg/d
		Quetiapine: 8/60 (13.33)			Quetiapine: 600–750 mg/d
Li et al. (90)	264	**Insomnia**			
		Risperidone: 18/134 (13.43)			Risperidone: 2–6 mg/d
		Blonanserin: 24/130 (18.46)			Blonanserin: 8–24 mg/d
Lin et al. (91)	92	**Somnolence**	**Reduced duration of sleep**		
		Amisulpride: 6/46 (13.04)	Amisulpride: 10/46 (21.74)		Amisulpride: 800 mg/d
		Amisulpride +sulpiride: 9/46 (19.56)	Amisulpride +sulpiride: 12/46 (26.07)		Amisulpride + sulpiride: 400 mg + 800 mg sulpirida/d
Lieberman et al. (92)	1,460	**Insomnia**	**Somnolence**	**Sedation**	
		Olanzapine: 55/336 (16.37)	Olanzapine: 104/336 (30.95)	Olanzapine: 7/336 (2.08)	Olanzapine: 7.5–30 mg/d
		Quetiapine: 62/337 (18.40)	Quetiapine: 103/337 (30.56)	Quetiapine: 9/337 (2.67)	Quetiapine: 200–800 mg/d
		Risperidone: 83/341 (24.34)	Risperidone: 96/341 (28.15)	Risperidone: 3/341 (0.88)	Risperidone: 1.5–6 mg/d
		Ziprasidone: 56/185 (30.27)	Ziprasidone: 45/185 (24.32)	Ziprasidone: 0/185 (0)	Ziprasidone: 40–160 mg/d
		Perphenazine: 66/261 (25.29)	Perphenazine: 74/261 (28.35)	Perphenazine: 7/261 (2.68)	Perphenazine: 8–32 mg/d
Loebel et al. (93)	361	**Insomnia**	**Somnolence**		
		Quetiapine XR: 5/119 (4.20)	Quetiapine XR: 16/119 (13.44)		Quetiapine XR: 200–800 mg/d
		Lurasidone: 8/121 (6.61)	Lurasidona: 8/121 (6.61)		Lurasidone: 40–160 mg/d
		Placebo: 11/121 (9.09)	Placebo: 1/121 (0.82)		Placebo
Loebel et al. (94)	486	**Somnolence**			
		Quetiapine XR: 16/119 (4.20)			Quetiapine XR: 600 mg/d
		Lurasidone 80 mg: 5/125 (4)			Lurasidone: 80 mg or 160 mg/d
		Lurasidone 160 mg: 8/121 (6.61)			
		Placebo: 1/121 (0.83)			Placebo: Equivalent dose
Luthringer et al. (95)	42	**Somnolence**			
		Paliperidone ER: 1/21 (4.76)			Paliperidone ER: 9 mg/d
		Placebo: 2/21 (9.52)			Placebo: 2/21 (9.52)
Marder et al. (96)	439	**Insomnia**	**Somnolence**		
		Olanzapine: 9/109 (8.25)	Olanzapine: 30/109 (27.52)		Olanzapine: 10 mg/d
		Paliperidone ER 6 mg: 16/112 (14.28)	Paliperidone ER 6 mg: 15/112 (13.39)		Paliperidone ER: 6 mg, or 12 mg/d
		Paliperidone ER 12 mg: 11/112 (9.82)	Paliperidone ER 12 mg: 15/112 (13.39)		
		Placebo: 13/106 (12.26)	Placebo: 14/106 (13.21)		Placebo: Equivalent dose
Meltzer et al. (97)	475	**Insomnia**	**Somnolence**	**Sedation**	
		Olanzapine: 11/122 (9.01)	Olanzapine: 13/122 (10.66)	Olanzapina: 18/122 (14.75)	Olanzapine: 15 mg/d
		Lurasidone 40 mg: 15/119 (12.60)	Lurasidone 40 mg: 12/119 (10.08)	Lurasidone 40 mg: 11/119 (9.24)	Lurasidone: 40 mg or 120 mg/d
		Lurasidone 120 mg: 18/118 (15.25)	Lurasidone 120 mg: 14/118 (11.86)	Lurasidone 120 mg: 16/118 (13.56)	
		Placebo: 5/116 (4.31)	Placebo: 13/116 (11.21)	Placebo: 4/116 (3.45)	Placebo
Meltzer et al. (98)	622	**Insomnia**	**Sedation**		
		Aripiprazole 441 mg: 20/207 (9.66)^*^	Aripiprazole 441 mg: 4/207 (1.93)^*^		Aripiprazole: 441 mg and 882 mg/monthly
		Aripiprazole 882 mg: 25/208 (12.02)^*^	Aripiprazole 882 mg: 5/208 (2.40)^*^		
		Placebo: 24/207 (11.59)^*^	Placebo: 3/207 (1.45)^*^		Placebo: Intralipid injection
Naber et al. (99)	250	**Insomnia**			
		Aripiprazole LAI: 4/132 (3.03)^*^			Aripiprazole LAI: 400 mg/month
		Paliperidone LAI: 8/118 (6.78)^*^			Paliperidone LAI: 50–150 mg/month
Nakamura et al. (100)	180	**Insomnia**	**Somnolence**	**Sedation**	
		Lurasidone: 9/90 (10)	Lurasidone: 10/90 (11.11)	Lurasidone: 9/90 (10)	Lurasidone: 80 mg/d
		Placebo: 3/90 (3.33)	Placebo: 3/90 (3.33)	Placebo: 4/90 (4.44)	Placebo: Equivalent dose
Nakamura et al. (101)	38	**Insomnia**	**Somnolence**		
		Cariprazine 3 mg/día: 2/11 (18.18)	Cariprazine 3 mg/día: 0/11 (0)		Cariprazine 3 mg, 6 mg, or 9 mg/d
		Cariprazine 6 mg/día: 4/16 (25.0)	Cariprazine 6 mg/día: 3/16 (18.75)		
		Cariprazine 9 mg/día: 2/11 (18.18)	Cariprazine 9 mg/día: 1/11 (9.09)		
Nasser et al. (102)	235	**Insomnia**	**Somnolence**		
		Risperidone LAI: 3/117 (2.56)^*^	Risperidone LAI: 5/117 (4.27)^*^		Risperidone LAI: 90 mg/month
					Risperidone LAI: 120 mg/month
		Placebo: 7/118 (5.93)^*^	Placebo: 0/118 (0)^*^		Placebo
Németh et al. (103)	460	**Insomnia**	**Somnolence**		
		Risperidone: 23/230 (10.0)	Risperidone: 13/230 (5.65)		Risperidone: 3–6 mg/d
		Cariprazine: 21/230 (9.13)	Cariprazine: 9/230 (3.91)		Cariprazine: 3–6 mg/d
Ogasa et al. (104)	149	**Insomnia**	**Somnolence**	**Sedation**	
		Lurasidone 40 mg: 3/50 (6)	Lurasidone 40 mg: 4/50 (8)	Lurasidone 40 mg: 9/50 (18)	Lurasidone: 40 mg or 120 mg/d
		Lurasidone 120 mg: 4/49 (8.16)	Lurasidone 120 mg: 5/49 (10.20)	Lurasidone 120 mg: 7/49 (14.29)	
		Placebo: 0/50 (0)	Placebo: 2/50 (4)	Placebo: 5/50 (10)	Placebo
Olié et al. (105)	123	**Insomnia**	**Somnolence**		
		Ziprasidone: 10/60 (16.67)	Ziprasidone: 2/60 (3.33)		Ziprasidone: 40–80 mg/d BID
		Amisulpride: 9/63 (14.28)	Amisulpride: 6/63 (9.52)		Amisulpride: 50–100 mg/d BID
Pandina et al. (106)	1,214	**Insomnia**			
		Risperidone LAI: 41/608 (6.74)^*^			Risperidone LAI: 25, 37.5, or 50 mg/2 weeks
		Paliperidone LAI: 26/606 (4.29)^*^			Paliperidone: 50 mg eq, 100 mg Eq, or 150 mg Eq/month
Pigott et al. (107)	306	**Insomnia**			
		Aripiprazole: 65/153 (42.48)			Aripiprazole: 15 mg/d
		Placebo: 61/153 (39.87)			Placebo: Equivalent dose
Potkin et al. (108)	121	**Insomnia**	**Somnolence**		
		Risperidone: 13/59 (22.03)	Risperidone: 9/59 (15.25)		Risperidone: 3 mg/d
		Asenapine: 11/59 (18.64)	Asenapine: 11/59 (18.64)		Asenapine: 5 mg/d
		Placebo: 8/62 (12.90)	Placebo: 8/62 (12.90)		Placebo: 1 tablet BID
Potkin et al. (109)	353	**Insomnia**	**Somnolence**	**Sedation**	
		Haloperidol: 10/72 (13.89)	Haloperidol: 9/72 (12.5)	Haloperidol: 14/72 (19.44)	Haloperidol: 10 mg/d
		Lurasidone 20 mg: 5/71 (7.04)	Lurasidone 20 mg: 4/71 (5.63)	Lurasidone 20 mg: 7/71 (9.85)	Lurasidone: 20 mg, 40 mg, or 80 mg
		Lurasidone 40 mg: 5/67 (7.46)	Lurasidone 40 mg: 4/67 (5.97)	Lurasidone 40 mg: 11/67 (16.42)	
		Lurasidone 80 mg: 4/71 (5.63)	Lurasidone 80 mg: 8/71 (11.27)	Lurasidone 80 mg: 14/71 (19.72)	
		Placebo: 4/72 (5.56)	Placebo: 4/72 (5.56)	Placebo: 6/72 (8.33)	Placebo: Equivalent dose
Purdon et al. (110)	25	**Somnolence**			
		Quetiapine: 1/13 (7.69)			Quetiapine: 300–600 mg/d
		Haloperidol: 1/12 (8.33)			Haloperidol: 10–20 mg/d
Riedel et al. (111)	19	**Insomnia**			
		Quetiapine: 6/9 (66.67)			Quetiapine: 400–800 mg/d
		Risperidone: 3/10 (30.0)			Risperidone: 4–8 mg/d
Riedel et al. (112)	65	**Somnolence**			
		Quetiapine IR: 2/34 (5.88)			Quetiapine IR: 400–750 mg/d
		Quetiapine XR: 1/31 (3.22)			Quetiapine XR: 400–750 mg/d
Savitz et al. (113)	1,016	**Insomnia**	**Somnolence**		
		Paliperidone 1st month: 24/512 (4.68)^*^	Paliperidone 1st month: 5/512 (0.97)^*^		Paliperidone: 50–150 mg Eq. per month
		Paliperidone 3rd month: 16/504 (3.17)^*^	Paliperidone 3rd month: 5/504 (0.99)^*^		Paliperidone: 175–525 mg Eq per three months
Sacchetti et al. (114)	146	**Insomnia**	**Somnolence**		
		Ziprasidone: 7/73 (9.58)	Ziprasidone: 3/73 (4.11)		Ziprasidone: 80–160 mg/d
		Clozapine: 2/73 (2.74)	Clozapine: 17/73 (23.29)		Clozapine: 250–600 mg/d
Shen et al. (115)	154	**Insomnia**	**Somnolence**	**Sedation**	
		Olanzapine: 3/77 (3.89)	Olanzapine: 8/77 (10.39)	Olanzapine: 15/77 (19.48)	Olanzapine: 15 mg/d
		Placebo: 5/77 (6.49)	Placebo: 4/77 (5.19)	Placebo: 7/77 (9.09)	Placebo: Equivalent dose
Sirota et al. (116)	40	**Insomnia**			
		Olanzapine: 6/21 (28.57)			Olanzapine: 5–20 mg/d
		Quetiapine: 6/19 (31.57)			Quetiapine: 200–800 mg/d
Small et al. (117)	286	**Insomnia**	**Somnolence**		
		Quetiapine ≤ 750 mg: 10/96–10.42	Quetiapine ≤ 750 mg: 24/96 (25)		Quetiapine ≤ 750 mg
		Quetiapine ≤ 250 mg: 12/94 (12.76)	Quetiapine ≤ 250 mg 18/94 (19.15)		Quetiapine ≤ 250 mg
		Placebo: 18/96 (18.75)	Placebo: 14/96 (14.58)		Placebo
Smith et al. (118)	69	**Insomnia**	**Somnolence**		
		Quetiapine al 2nd day: 6/24 (25)	Quetiapine al 2nd day: 2/24 (8.33)		Quetiapine 400 mg in 2 days
		Quetiapine al 3rd day: 1/21 (4.76)	Quetiapine al 3rd day: 3/21 (14.28)		Quetiapine 400 mg in 3 days
		Quetiapine al 5th day: 6/22 (27.27)	Quetiapine al 5th day: 3/22 (13.63)		Quetiapine 400 mg in 5 days
Stroup et al. (119)	444	**Insomnio**	**Somnolencia**	**Sedation**	
		Olanzapine: 13/108 (12.04)	Olanzapine: 28/108 (25.93)	Olanzapine: 0/108 (0)	Olanzapine: 7.5–30 mg/d
		Quetiapine: 16/95 (16.84)	Quetiapine: 23/95 (24.21)	Quetiapine: 1/95 (1.05)	Quetiapine: 200–800 mg/d
		Risperidone: 23/104 (22.12)	Risperidone: 22/104 (21.15)	Risperidone: 1/104 (0.96)	Risperidone: 1.5–6 mg/d
		Ziprasidone: 31/137 (22.63)	Ziprasidone: 13/137 (9.49)	Ziprasidone: 1/137 (0.73)	Ziprasidone: 40–160 mg/d
Stroup et al. (120)	115	**Insomnia**			
		Olanzapine: 4/39 (10.26)			Olanzapina: 7.5–30 mg/d
		Quetiapine: 7/38 (18.42)			Quetiapina: 200–800 mg/d
		Risperidone: 6/38 (15.78)			Risperidona: 1.5–6 mg/d
Takekita et al. (121)	100	**Insomnia**	**Somnolence**		
		Aripiprazole: 26/49 (53.06)	Aripiprazole: 4/49 (8.16)		Aripiprazole: 3–30 mg/d
		Perospirone: 18/51 (35.29)	Perospirone: 4/51 (7.84)		Perospirone: 8–48 mg/d
Takeuchi et al. (122)	677	**Insomnia**	**Somnolence**		
		Risperidone: 65/173 (37.57)	Risperidone: 50/173 (28.90)		Risperidone: 1.5–6 mg/d
		Olanzapine: 51/169 (30.18)	Olanzapine: 61/169 (36.09)		Olanzapine: 7.5–30 mg/d
		Risperidone BID: 66/168 (39.28)	Risperidone BID: 65/168 (38.69)		Risperidone BID: 1.5–6 mg/d
		Olanzapine BID: 50/167 (29.94)	Olanzapine BID: 80/167 (47.90)		Olanzapine BID: 7.5–30 mg/d
Tandon et al. (123)	285	**Insomnia**	**Sedation**		
		Lurasidone: 3/144 (2.08)	Lurasidona: 1/144 (0.69)		Lurasidone: 40–80 mg/d
		Placebo: 4/141 (10.52)	Placebo: 0/141 (0)		Placebo: Equivalent dose
Tzimos et al. (124)	114	**Insomnio**	**Somnolence**		
		Paliperidone ER: 7/76 (9.21)	Paliperidone ER: 7/76 (9.21)		Paliperidone ER: 3–12 mg/d
		Placebo: 4/38 (10.52)	Placebo: 2/38 (5.26)		Placebo: Equivalent dose
Weiden et al. (125)	301	**Insomnia**	**Somnolence**		
		Iloperidone: 3/151 (1.98)	Iloperidone: 2/151 (1.32)		Iloperidone: 8–24 mg/d
		Placebo: 7/150 (4.67)	Placebo: 0/150 (0)		Placebo: Equivalent dose
Zhong (126)	672	**Somnolence**			
		Quetiapine: 89/338 (26.33)			Quetiapine: 200–800 mg/d
		Risperidone: 66/334 (19.76)			Risperidone: 2–8 mg/d

^*^Injectable antipsychotic.

IR, Immediate release; XR, extended release; ER, extended release; BID, Twice a day; LD, Low dose; MD, Median dose; HD, High dose; mg/d, Milligrams per day; mg Eq, Milligram per equivalent.

**Table 2:** Operational definitions of insomnia, somnolence, and sedation.

**Variable**	**Operational definitions**	**Outcome**
Insomnia (127, 128)	Sleep disturbance characterized by difficulty initiating or maintaining sleep, or nonrestorative sleep that occurs at least 3 nights per week (time measured in hours).	Frequency and percentage of patients with insomnia who received antipsychotic treatment.
Somnolence (134)	Total time in hours scored as complaint of excessive sleepiness or fatigue during waking time or, less commonly, a long sleep period.	Frequency and percentage of patients with somnolence who received antipsychotic treatment.
Sedation (137)	Total time in hours scored as reduction of irritability or agitation and permitting a person sleep by administration of sedative drugs.	Frequency and percentage of patients with sedation who received antipsychotic treatment.

These adverse effects according to the level of severity can be mild, moderate or severe.

The AP given in randomized clinical trials (RCT) were the following: amisulpride, aripiprazole, aripiprazole LAI (every 28 days), asenapine, blonanserin, brexpiprazole, cariprazine, chlorpromazine, clozapine, fluphenazine, flupentixol, haloperidol, iloperidone, lurasidone, olanzapine, olanzapine LAI (monthly), paliperidone, paliperidone LAI (monthly), perphenazine, perospirone, quetiapine, risperidone, risperidone LAI (every 15 days), sertindole, ziprasidone, zotepine, and placebo.

**Figure 2:**
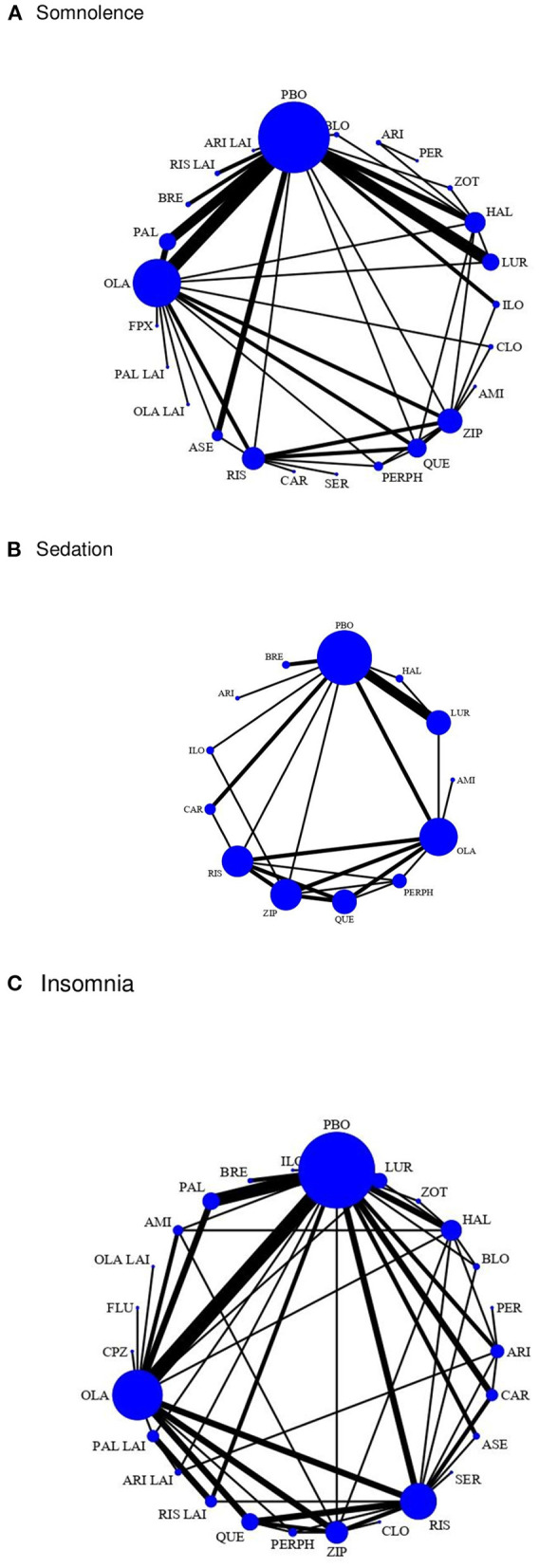
The network of comparisons of the antipsychotics included in this study for somnolence, sedation, and insomnia. **(A)** Somnolence. **(B)** Sedation. **(C)** Insomnia. The width of the lines is proportional to the number of trials comparing each treatment pair and the size of each node is proportional to the number of randomized participants. The nodes represent the included interventions, and their size is proportional to the sample size. The thickness of the edges connecting the nodes reflects the number of trials included in the given comparison. The thickness of the edges connecting the nodes reflects the number of trials included in the given comparison. AMI, amisulpride; ASE, asenapine; ARI, aripiprazole; ARI LAI, aripiprazole long-acting injection; BLO, blonanserin; BRE, brexpiprazole; CAR, cariprazine; CLO, clozapine; CPZ, chlorpromazine; FLU, fluphenazine; FPX, flupentixol; HAL, haloperidol; ILO, iloperidone; LUR, lurasidone; OLA, olanzapine; OLA LAI, olanzapine long-acting injection; PAL, paliperidone; PAL LAI, paliperidone long-acting injectable; PBO, placebo; PER, perospirone; PERPH, perphenazine; QUE, quetiapine; RIS, risperidone; RIS LAI, risperidone long-acting injection; SER, sertindole; ZIP, ziprasidone; ZOT, zotepine.

It is important to note that in most of the studies included in the review, antipsychotics were administered orally (*n* = 72, 82.76%), or they were administered intramuscularly, the safety and efficacy of antipsychotics were compared, an antipsychotic tablet vs. the injectable presentation (*n* = 15, 17.24%). Of the total number of RCTs included in the systematic review, 61 were included in the quantitative analysis phase of the network meta-analysis according to the criterion of having at least two treatment arms comparing different antipsychotics or comparing an antipsychotic with another placebo (see Figure 2).

Clinical observations, subjective reports or validated questionnaires were used to report adverse sleep effects in the clinical trials. There was a low percentage of studies in which the method of reporting these negative effects was unclear (*n* = 3, 3.45 %).

### Methodological quality of the studies

In the clinical trials included in the review, all evaluating the safety of one or more antipsychotics vs. another antipsychotic or placebo, the studies varied in reporting the most important methodological characteristics, including: random sequence generation, concealment of allocation, blinding of interventions, appropriate blinding, intention to treat, and description of loss to follow up. When evaluating the level of bias risk, it was observed that the unclear risk predominates, at the point of incomplete data of the results the bis risk is low, it is worth mentioning that in other types of bias risk is high. CONSORT evaluations of methodological quality are summarized in Table 3.

**Table 3:** Percentage distribution of scoring for each quality item in CONSORT checklist for articles included.

**CONSORT ítem**	**Appropriate**	**No description**	**Do not apply**
	***n* (%)**	***n* (%)**	***n* (%)**
Title	46 (52.27)	42 (47.73)	–
**Method**			
Trial design	57 (64.77)	31 (35.23)	–
Participants	86 (98.85)	1 (1.14)	–
Interventions	62 (70.45)	26 (29.55)	–
Objective	37 (42.05)	51 (57.95)	–
Primary and secondary outcomes	82 (93.18)	6 (6.82)	–
Randomization	39 (44.32)	49 (55.68)	–
Blinding	49 (55.68)	30 (34.09)	9 (10.23)
Statistical methods	86 (98.85)	1 (1.14)	–
**Results**			
Number of randomized participants	50 (56.82)	38 (43.18)	–
Recruitment of participants	55 (62.50)	32 (36.36)	1 (1.14)
Analyzed data	77 (87.50)	11 (12.50)	–
Results	60 (68.18)	28 (31.82)	–
Damage	87 (100)	0	–
Conclusions	61 (69.32)	27 (30.68)	–
Trial registration	46 (52.27)	42 (47.73)	–
Funding	82 (93.18)	6 (6.82)	–

Bias risk assessment was done in the following order: random sequence generation, allocation concealment, blinding of participants and personnel, blinding of outcome assessors, incomplete outcome data, selective outcome reporting, and other biases.

When reviewing the bias risk in the 87 articles (40–126), it was found there was low or moderate risk in most clinical trials, 36 studies (41.38%) reported adequate random sequence generation and in 39 studies (44.83%) the bias risk was unclear. Concerning participants and health personnel blinding, 37 trials (42.53%) had a low bias risk and 38 were unclear (43.68%). In 15 studies (17.24%) it was reported that the allocation concealment was carried out by a person independent of the research team and in 45 (51.72%) it was not clear. In the section on blinding of outcome assessors, 20 studies (22.98%) had a low bias risk and in 54 (62.07%) the risk was unclear. Intention-to-treat analysis was applied in 58 studies (66.67%), in the rest of the studies it is not mentioned, or it is unclear how the analysis of missing data was done. Regarding the selective reporting of results, 95.39% (*n* = 83) of the articles had a low or unclear level of bias (see Risk table of bias, of the Supplementary material).

A total of 25 CONSORT items were evaluated in duplicate for each article, according to the response options: yes, no and not applicable. After calculating the frequencies and percentages of all the items, we found that 47 articles (53.41%) met the quality criteria in more than 50% and 41 (46.59%) met <50% of the checklist criteria of CONSORT, according to these results, the methodological quality in more than half of the articles was acceptable.

### Network meta-analysis results

We compared the safety on sleep of 22 orally administered AP and four injectable AP used for schizophrenia treatment, the established reference category being placebo. We reviewed 70 randomized clinical trials published between 1990 and 2021; 61 trials reported insomnia as an adverse effect (87.14%), 41 studies reported somnolence (58.57%), and 15 (21.42%) reported sedation related to antipsychotic use. Figure 2 shows the network of comparisons of the antipsychotics included in this study for insomnia, somnolence, and sedation. The size of the nodes indicates that placebo is the most common comparator across trials, we weight all edges connecting placebo to an active treatment according to the number of trials.

Network consistency was examined using the loop-specific approach. Inconsistency factors were not found to be significant, although this cannot be taken as evidence of the absence of inconsistency. This is due to low power in some of the loops, especially in the presence of large heterogeneity in pairwise comparisons. On the other hand, when performing the analysis of global inconsistency in the network, there was no inconsistency in the case of insomnia (x2 = 36.85, df = 39, *p* = 0.57), somnolence (x2 = 34.10, df = 22, *p* = 0.05) and sedation (x2 = 7.46, df = 9, *p* = 0.59).

In the SUCRA analysis of studies reporting insomnia, chlorpromazine clearly ranked as the safest antipsychotic and the least likely to cause difficulty sleeping (Pr = 57%, SUCRA = 0.9), followed by clozapine (Pr = 20%, SUCRA = 0.8) and iloperidone (Pr = 16.9%, SUCRA = 0.8). The multivariate meta-regression analysis performed to examine the main nodes of the network taking placebo as the reference category, found that ziprasidone increases the risk of insomnia, OR= 1.56 (95% CrI: 1.18–2.06) while this risk is reduced with the use of olanzapine OR= 0.67 (95% CrI: 0.52–0.85), see Table 4.

**Table 4:** Antipsychotic multivariate meta-regression results and adverse effects on sleep.

**Treatment**	**Insomnia**			**Somnolence**			**Sedation**		
	**OR**	**95% CrI**	***P-*value**	**OR**	**95% CrI**	***P*-value**	**OR**	**95% CrI**	***P-*value**
Amisulpride	1.14	0.72, 1.81	0.565	5.47	0.89, 33.46	0.066	0.12	0.01, 2.19	0.151
Aripiprazole	1.17	0.90, 1.51	0.236	1.25	0.51, 3.08	0.632	6.69	0.34, 131.48	0.211
Aripiprazole LAI	0.92	0.57, 1.48	0.736	1.67	0.36, 7.89	0.514	–	–	–
Asenapine	0.66	0.38, 1.12	0.125	1.03	0.56, 1.92	0.916	–	–	–
Blonanserin	1.59	0.87, 2.88	0.126	2.75	0.60, 12.64	0.193	–	–	–
Brexpiprazole	0.95	0.61, 1.50	0.853	1.22	0.47, 3.12	0.683	3.41	0.93, 12.54	0.065
Cariprazine	1.29	0.92, 1.80	0.135	1.42	0.45, 4.52	0.552	1.38	0.75, 2.55	0.305
Chlorpromazine	0.2	0.04, 1.08	0.061	–	–	—	–	–	–
Clozapine	0.41	0.81, 2.11	0.29	14.29	4.58, 44.55	0.000^*^	–	–	–
Fluphenazine	10.84	0.57, 204.0	0.111	–	–	–	–	–	–
Flupentixol	–	–	–	1.41	0.10, 19.27	0.795	–	–	–
Haloperidol	1.21	0.91, 1.60	0.193	1.9	1.12, 3.22	0.017^*^	2.61	1.14, 5.99	0.024^*^
Iloperidone	0.41	0.11, 1.63	0.208	1.62	0.59, 4.48	0.351	1.46	0.76, 2.81	0.255
Lurasidone	0.95	0.61, 1.49	0.828	2.25	1.28, 3.97	0.005^*^	2.63	1.57, 4.41	0.000^*^
Olanzapine	0.67	0.52, 0.85	0.001^*^	2.61	1.82, 3.75	0.000^*^	3.26	1.87, 5.69	0.000^*^
Olanzapine LAI	0.74	0.40, 1.36	0.341	2.34	0.96, 5.72	0.062	–	–	–
Paliperidone	0.81	0.57, 1.17	0.264	1.25	0.74, 2.11	0.399	–	–	–
Paliperidone LAI	0.96	0.63, 1.45	0.854	12.18	1.15, 128.30	0.037^*^	–	–	–
Perphenazine	1.21	0.83, 1.77	0.314	2.26	1.17, 4.39	0.015^*^	5.33	1.92, 14.83	0.001^*^
Perospirone	0.56	0.24, 1.31	0.184	1.19	0.20, 7.18	0.846	–	–	–
Quetiapine	0.83	0.60, 1.16	0.292	2.72	1.57, 4.71	0.000^*^	5.14	2.07, 12.73	0.000^*^
Risperidone	1.16	0.92, 1.47	0.21	2.09	1.24, 3.51	0.005^*^	2.41	1.21, 4.80	0.012^*^
Risperidone LAI	1.08	0.74, 1.58	0.669	4.42	1.21, 16.08	0.024^*^	–	–	–
Sertindole	0.84	0.32, 2.21	0.728	2.2	0.60, 8.11	0.236	–	–	–
Ziprasidone	1.56	1.18, 2.06	0.002^*^	1.79	1.06, 3.02	0.029^*^	3.64	1.92, 6.91	0.000^*^
Zotepine	0.83	0.34, 2.04	0.686	4.3	1.91, 9.66	0.000^*^	–	–	–

^*^p < 0.05.

In the case of sedation, amisulpride was the safest antipsychotic (Pr = 89.9%, SUCRA = 1.0). The results of the multivariate meta -regression indicated that haloperidol increased the risk of sedation OR = 2.61 (95% CrI: 1.14–5.99), the same occurred with lurasidone OR = 2.63 (95% CrI: 1.57–4.41), olanzapine OR = 3.25 (95% CrI: 1.87–5.69), perphenazine OR = 5.33 (95% CrI: 1.92–14.83), quetiapine OR = 5.14 (95% CrI: 2.07–12.72), risperidone OR = 2.41 (95% CrI: 1.21–4.80) and ziprasidone OR = 3.64 (95% CrI: 1.92–6.91), while with the rest of the antipsychotics there were no statistically significant associations.

Regarding somnolence, fluphenazine (Pr = 26%, SUCRA = 0.6) and perospirone (Pr = 22.5%, SUCRA = 0.7) were the safest medications; in the multivariate meta-regression we found that clozapine OR = 14.29 (95% CrI: 4.58–44.55), haloperidol OR = 1.90 (95% CrI: 1.12–3.22), lurasidone OR = 2.25 (95% CrI: 1.28–3.97), olanzapine OR = 2.61 (95% CrI: 1.82–3.75), paliperidone LAI OR = 12.18 (95% CrI: 1.15–128.30), perphenazine OR = 2.26 (95% CrI: 1.17 – 4.39), quetiapine OR = 2.72 (95% CrI: 1.57–4.71), risperidone OR = 2.09 (95% CrI: 1.24–3.51), risperidone LAI OR= 4.42 (95% CrI: 1.21–16.08), ziprasidone OR = 1.79 (95% CrI: 1.06–3.02) and zotepine OR = 4.30 (95% CrI: 1.91–9.66) increased the risk of somnolence. Finally, in the geometry of the network it is observed that the size of the nodes corresponds to the number of RCTs that studied the adverse effects of antipsychotics on sleep, the directly comparable antipsychotics are linked with a line, and the thickness of each line corresponds to the inverse variance of the number of direct comparisons [see The ranking of antipsychotics (SUCRA) table, in the Supplementary material].

## Discussion

The original objective of this research was to carry out a systematic review and meta-analysis of the adverse effects on sleep in patients over 18 with schizophrenia receiving antipsychotic treatment. The most frequently reported sleep effects in randomized clinical trials were insomnia, somnolence, and sedation. This is consistent with studies describing DRA effects on sleep. Waite et al. (127) reported in a systematic review that 50% of patients with psychosis presented insomnia. This sleep problem improved considerably and modest changes in psychotic symptoms were observed when receiving some psychological intervention. In another study, Thompson et al. (128) conducted a review evaluating the benefits and adverse effects of atypical antipsychotics, finding that there was no clinical difference in insomnia administering quetiapine or placebo. However, they included only 13 studies, so the evidence quality was low.

When evaluating the quality of the studies reviewed using the CONSORT checklist, we found that more than half of the articles (53.41%) showed acceptable methodological quality. In most of the included studies (*n* = 87) bias risk was classified as low and unclear. For example, in random sequence generation, the sum of the percentage of low and unclear risk reached 86.21%, in allocation concealment it reached 68.97%, in blinding of participants and personnel it showed 86.21%, in the blinding of result evaluators, it was 85.06%, in the incomplete data it reached 87.36%, in the selective reporting of results it reached 95.40% and in other biases it was 62.07%.

We also found that FGAs and SGAs have adverse effects on sleep and are less safe than no antipsychotic for the treatment of symptoms in schizophrenia. The effect sizes are clinically meaningful and comparable to previous findings. In contrast to our results, in a head-to-head comparison study of 37 antipsychotics, zuclopenthixol was found to rank first in association with sedation and somnolence among drugs reported to FAERS (129), and are consistent with the large-scale network meta-analysis in which zuclopenthixol stood out among 32 antipsychotics and their found that older antipsychotics were associated with prolactin elevation and more extrapiramidal side effects, whereas newer antipsychotics were associated with more sedation and weight gain (12). In addition, Kishi et al. (130) examined the safety of antipsychotic use in schizophrenia using only randomized trials conducted in Japan. They found that asenapine, lurasidone, olanzapine, perospirone, quetiapine, and mosapramine were associated with a higher incidence of somnolence. Clozapine was associated with a lower incidence of insomnia compared with placebo.

The safety ranking of different AP, based on the SUCRA calculation regarding insomnia, indicated that probability of presenting insomnia as an adverse effect, chlorpromazine and clozapine were the safest AP. Participants receiving amisulpride were less likely to experience sedation, while those receiving flupentizole and perospirone reported less sleepiness. In contrast, the AP most likely to affect sleep were ziprasidone and fluphenazine (insomnia), perphenazine and quetiapine (sedation), and clozapine and zotepine (somnolence).

Our results show that olanzapine reduced insomnia risk by 22% when compared to placebo. This AP has been shown to have a positive effect on sleep efficiency, slow wave sleep and REM sleep (131, 132). Ziprasidone increased the risk of insomnia by 1.58 times (*p* < 0.01). There is evidence that the three most frequent adverse events associated with the use of ziprasidone are: insomnia (21–42%), somnolence (14–26%), and anxiety (19–21%) (133).

On the other hand, we found that haloperidol, lurasidone, olanzapine, perphenazine, quetiapine, risperidone, zotepine, and ziprasidone caused sedation and somnolence as adverse sleep effects. Those that caused greater sedation were perphenazine (OR= 5.33), quetiapine (OR= 5.14) and ziprasidone (OR= 3.64). Among the AP that caused more sedation were clozapine (OR = 14.42), zotepine (OR = 4.30), and quetiapine (OR = 2.77). All these values were statistically significant (*p* < 0.01).

Our results are similar to other previous studies. Fang et al. (134) carried out a systematic review in which they considered the Absolute Risk Increase and the NNH to classify AP by the level of somnolence. Clozapine had a high level of somnolence; olanzapine, perphenazine, quetiapine, risperidone, and ziprasidone had a moderate level of somnolence, while those that produced low somnolence were aripiprazole, haloperidol, lurasidone, asenapine, paliperidone, and cariprazine.

Ziprasidone is generally better tolerated on sleep compared to more sedating AP, because it has a shorter duration of action (2–4 h) (135).

Adverse effects on sleep related to antipsychotic drugs use generally have a negative impact on functionality and social interaction, preventing patients with schizophrenia from benefiting from psychiatric rehabilitation and other treatments. For example, sedation and somnolence would negatively increase negative feelings and attitudes toward taking AP, making it difficult for patients to reintegrate into society (136–138). In contrast, AP with mild or no adverse effects on sleep would improve treatment compliance and long-term efficacy (130).

Regarding the limitations, it is important to mention that some articles could not be retrieved in full, despite performing the search in different bibliographic databases, for this reason they were not included in the analysis. Search terms were limited to thesaurus Mesh and DeCS, it cannot be assumed that all the articles and areas in the health field that address the safety of AP have been covered. The available evidence was limited for some comparisons, especially for the older antipsychotics and those of recent appearance. We missed some RCT that were published before 1990, however in 1994, might have conditioned some differences between oldest and newer studies. For example, the change from DSM-III-TR to DSM-IV (139).

All articles were written in English, and despite the search of journal databases in Spanish, no article was found that met our inclusion criteria. Overall, we identified a wide variety of doses and treatment durations in the intervention groups, many different groups of comparators, and variations in the diagnostic criteria and clinical characteristics of patients with schizophrenia, which increased the heterogeneity between trials. Despite the abundance of safety variables that we planned to assess, the available information in each clinical trial was usually reported using different methods, making meta-analyses challenging. Nevertheless, it is possible to draw conclusions from direct and indirect comparisons.

On the other hand, articles that studied AP adverse effects in patients with other psychotic disorders (e.g., schizoaffective disorder, schizophreniform disorder, brief psychotic disorder, and delusional disorder) were not included. In addition, there is the heterogeneity shown by the articles found by age range, the duration of the studies, the size of the participant samples, as well as the results reported.

In terms of clinical practice, our review provides information on the adverse effects of antipsychotics on sleep. This may improve the decision-making process when prescribing AP treatment for each patient, taking into account the safety profile and which of these AP exacerbate or ameliorate sleep problems. Regarding the implications for research, we show that the available evidence on sleep effects of most AP is still insufficient, and further studies are needed in this population. We suggest better reporting of methodological features in primary studies (e.g., description of allocation concealment procedure, blinding of outcome assessors, RCT registration).

In order to better elucidate the safety of AP on sleep, new studies on larger populations are needed. Furthermore, there was a degree of uncertainty in identifying which sleep-related adverse effects were linked to antipsychotic drugs, as this was generally determined by the staff members' understanding, study investigators or by spontaneous reports of patients, especially in articles that were published between 1990 and 2000, because the conceptual definitions or methodological quality of ADRs is not well-described. In this way, health care decision makers can consider the safest therapeutic option at a lower cost, which could be an alternative to be implemented in adults with schizophrenia.

## Conclusion

The antipsychotics with the least adverse effects on sleep were olanzapine, chlorpromazine, and clozapine for insomnia; flupentixol and perospirone for somnolence; amisulpride had a lower risk of sedation.

In contrast, those with a less secure profile were ziprasidone, blonanserin, and fluphenazine (insomnia); clozapine, zotepine, and paliperidone LAI (somnolence); perphenazine and quetiapine (sedation).

These results are mainly referable to oral AP; in the case of injectable AP, it is necessary to expand the scientific evidence, route of administration should be considered in the analysis. We also suggest carrying out a systematic review on injectables AP effects on sleep.

Adverse effects on sleep may not always be desirable, but they are often difficult to avoid in psychiatric patients. Some of the most AP have unique benefits that may justify their use. Dosing according to the clinical response may improve tolerability, adverse effects do not get in the way.

## Data availability statement

The original contributions presented in the study are included in the article/Supplementary material, further inquiries can be directed to the corresponding author.

## Author contributions

YV contributed to the conception and design of the study, organized the database, and performed the statistical analysis. RS-A contributed to the conception and design of the study, organized the database, and wrote the first draft of the manuscript. VV, DV, and GN contributed to the conception and design of the study. RE wrote sections of the manuscript. All authors contributed to the article and approved the submitted version.

## Glossary

PROSPERO: International prospective register of systematic reviews
REM: Rapid eyes movement
AP: Antipsychotics
DRA: Dopamine receptor antagonists
FGAs: First Generation Antipsychotics
SGAs: Second Generation Antipsychotics
ADRs: Adverse Drug reactions
LAI: Long-action injectable
CONSORT: Consolidated Standards of Reporting Trials
NMA: Network meta-analysis
RCT: Randomized Clinical Trials
CrI: Credible Interval
CONACyT: Consejo Nacional de Ciencia y Tecnología
NNH: Number Needed to Harm
SUCRA: Surface under the cumulative ranking curve
MeSH: Medical subject headings terms
DeCS: Descriptores en Ciencias de la Salud
Pr: Probability
IR: Immediate release
XR: extended release
ER: extended release
BID: Twice a day
LD: Low dose
MD: Median dose
HD: High dose
mg/d: Milligrams per day
mg Eq: Milligram per equivalent
AMI: Amisulpride
ASE: Asenapine
ARI: Aripiprazole
ARI LAI: Aripiprazole Long-acting injection
BLO: Blonanserin
BRE: Brexpiprazole
CAR: Cariprazine
CLO: Clozapine
CPZ: Chlorpromazine
FLU: Fluphenazine
FPX: Flupentixol
HAL: Haloperidol
ILO: Iloperidone
LUR: Lurasidone
OLA: Olanzapine
OLA LAI: Olanzapine Long-acting injection
PAL: Paliperidone
PAL LAI: Paliperidone Long-acting injectable
PBO: Placebo
PER: Perospirone
PERPH: Perphenazine
QUE: Quetiapine
RIS: Risperidone
RIS LAI: Risperidone Long-acting injection
SER: Sertindole
ZIP: Ziprasidone
ZOT: Zotepine.
