# Supplementary material for: Adverse effects of antipsychotics on sleep in patients with schizophrenia. Systematic review and meta-analysis
Source: Front Psychiatry. 2023 Jun 27;14:1189768. doi: 10.3389/fpsyt.2023.1189768 (PMC10333591; doi:10.3389/fpsyt.2023.1189768)

Supplementary Material

Adverse effects of antipsychotics on sleep in patients with schizophrenia. Systematic review and meta-analysis.

**Yarmila Elena Valencia Carlo ^1^, Ricardo Arturo Saracco-Alvarez ^2*^, Verónica Angela Valencia Carlo ^3^, Daniela Vázquez Vega^4^, Guillermina Natera Rey^5^, Raul Ivan Escamilla Orozco^6^**

* Correspondence:

Ricardo Arturo Saracco-Alvarez

email: [dr_saracco@yahoo.com.mx](mailto:dr_saracco@yahoo.com.mx), [saracco@imp.edu.mx](mailto:saracco@imp.edu.mx)

# Supplementary Data

# In the paper, due to space limitation, we only showed a small portion of our results. In this supplementary material we present more quantitative results to support the findings of our study.

# Table S1 Extraction of data was directly from journal articles, it used to capture the data they then summarized or analyzed. In table S2 and Figure S1, show a summary of the risk of bias, some domains affect the risk of bias across outcomes in a study: e.g., sequence generation and allocation sequence concealment. Other domains, such as blinding and incomplete outcome data, may have different risks of bias for different outcomes within a study. Table S3 shows SUCRA values range from 0 to 100 %. They were used for more precise estimation of cumulative ranking probabilities of antipsychotics. In tables S4, S5 and S6 show the results of the local inconsistency, listing the size of differences for each treatment and the statistical test results. None of the treatments showed statistical significance, because inconsistency was found to be absent in both global and local tests, the consistency assumption was accepted. In the figures 2, 3, and 4 represent graphically the results of network rank test that is a presentation of the overall ranking and presents a single number associated with each treatment.

# Index of tables and figures

# Table S1. Data extraction table corresponding to the articles included in systematic review

# Table S2. Summary by type of risk of bias

# Table S3. Ranking of antipsychotics and adverse effects on sleep

# Table S4. Direct and indirect comparisons for insomnia network

# Table S5. Direct and indirect comparisons for somnolence network

# Table S6. Direct and indirect comparisons for sedation network

# Figure S1. Risk of bias for the selected studies

# Figure S2. Network range test antipsychotics and insomnia

# Figure S3. Network range test antipsychotics and somnolence

# Figure S4. Network range test antipsychotics and sedation

# Supplementary Figures and Tables

#

# Table S3. Ranking of antipsychotics (SUCRA) and adverse effects on sleep

|  | Insomnia | | | Somnolence | | | Sedation | | |
| --- | --- | --- | --- | --- | --- | --- | --- | --- | --- |
| Ranking of treatments | SUCRA | Probability  best (%) | Mean rank | SUCRA | Probability  best (%) | Mean rank | SUCRA | Probability  best (%) | Mean rank |
| Amisulpride  Aripiprazole  Aripiprazole LAI  Asenapine  Blonanserin  Brexpiprazole  Cariprazine  Chlorpromazine  Clozapine  Fluphenazine  Flupentixol  Haloperidol  Iloperidone  Lurasidone  Olanzapine  Olanzapine LAI  Paliperidone  Paliperidone LAI  Perphenazine  Perospirone  Quetiapine  Risperidone  Risperidone LAI  Sertindole  Ziprasidone  Zotepine | 0.4  0.3  0.5  0.8  0.2  0.5  0.2  0.9  0.8  0.1  ---  0.3  0.8  0.5  0.8  0.7  0.6  0.5  0.3  0.8  0.6  0.3  0.4  0.6  0.1  0.6 | 0.0  0.0  0.0  0.8  0.0  0.0  0.0  57.0  20.0  0.3  ---  0.0  16.9  0.0  0.0  0.2  0.0  0.0  0.0  3.1  0.0  0.0  0.0  0.7  0.0  1.0 | 17.2  18.2  12.3  6.8  22.2  13.2  20.1  2.4  6.1  24.6  ---  19.0  5.6  13.3  6.3  9.1  9.9  13.5  18.8  6.2  10.3  18.2  16.8  11.6  23.6  11.7 | 0.2  0.7  0.6  0.8  0.4  0.7  0.7  ---  0.0  ---  0.6  0.5  0.6  0.4  0.4  0.4  0.8  0.1  0.5  0.7  0.3  0.5  0.2  0.5  0.6  0.2 | 0.9  4.5  7.4  7.6  1.9  8.2  8.4  ---  0.0  ---  26.0  0.0  3.2  0.0  0.0  0.0  2.8  0.6  0.0  22.5  0.0  0.0  0.0  2.9  0.0  0.0 | 19.7  7.2  10.9  4.8  15.4  7.2  8.5  ---  24.1  ---  10.4  12.1  10.2  14.4  16.4  14.3  6.6  22.6  14.0  8.2  16.7  13.1  19.3  13.5  10.8  20.4 | 1.0  0.2  ---  ---  ---  0.4  0.8  ---  ---  ---  ---  0.5  0.7  0.5  0.4  ---  ---  ---  0.2  ---  0.2  0.5  ---  ---  0.3  --- | 89.9  1.7  ---  ---  ---  0.1  2.0  ---  ---  ---  ---  0.0  1.3  0.0  0.0  ---  ---  ---  0.0  ---  0.0  0.0  ---  ---  0.0  ---- | 1.4  10.1  ---  ---  ---  8.6  3.8  ---  ---  ---  ---  7.2  4.0  7.2  8.7  ---  ---  ---  10.9  ---  10.8  6.8  ---  ---  9.2  --- |

# Sucra: Surface Under the Cumulative Ranking Curve

# Table S4. Direct and indirect comparisons for the insomnia network

|  | **Direct** | | **Indirect** | | **Difference** | | |
| --- | --- | --- | --- | --- | --- | --- | --- |
| **Comparison** | **Coefficient** | **Std. Err.** | **Coefficient** | **Std. Err.** | **Coefficient** | **Std. Err.** | **P-value** |
| Amisulpride vs placebo | -0.381 | 0.484 | 0.312 | 0.275 | -0.692 | 0.566 | 0.222 |
| Aripiprazole vs placebo | -0.089 | 0.192 | 0.374 | 0.181 | -0.464 | 0.263 | 0.077 |
| Aripiprazole LAI vs placebo | 0.126 | 0.364 | -0.247 | 0.325 | 0.373 | 0.488 | 0.445 |
| Asenapine vs placebo | -0.419 | 0.296 | -0.450 | 0.842 | 0.031 | 0.905 | 0.972 |
| Blonanserin vs placebo | - 4.73 | 0.606 | 0.620 | 0.351 | -0.620 | 0.701 | 0.376 |
| Cariprazine vs placebo | 0.294 | 0.211 | 0.174 | 0.300 | 0.121 | 0.370 | 0.745 |
| Haloperidol vs placebo | 0.099 | 0.310 | 0.212 | 0.163 | -0.113 | 0.352 | 0.748 |
| Lurasidone vs placebo | 0.118 | 0.247 | -1.099 | 0.622 | 1.218 | 0.672 | 0.070 |
| Olanzapine vs placebo | -0.254 | 0.163 | -0.577 | 0.179 | 0.323 | 0.242 | 0.184 |
| Paliperidone vs placebo | -0.145 | 0.196 | -0.598 | 0.489 | 0.452 | 0.523 | 0.387 |
| Paliperidone LAI vs placebo | -0.587 | 0.405 | 0.168 | 0.249 | -0.755 | 0.476 | 0.112 |
| Risperidone vs placebo | 0.422 | 0.242 | 0.060 | 0.139 | 0.361 | 0.279 | 0.197 |
| Risperidone LAI vs placebo | -0.110 | 0.346 | 0.170 | 0.233 | -0.281 | 0.417 | 0.501 |
| Ziprasidone vs placebo | 0.591 | 0.355 | 0.416 | 0.156 | 0.175 | 0.388 | 0.653 |
| Zotepine vs placebo | -0.210 | 0.553 | -0.130 | 0.817 | -0.080 | 0.987 | 0.935 |

# Table S5. Direct and indirect comparisons for the sedation network

|  | Direct | | Indirect | | Difference | | |
| --- | --- | --- | --- | --- | --- | --- | --- |
| Cariprazine vs Placebo | 0.326 | 0.329 | 0.259 | 1.426 | 0.067 | 1.492 | 0.964 |
| Haloperidol vs placebo | 0.977 | 0.520 | 0.908 | 0.998 | 0.068 | 1.206 | 0.955 |
| Iloperidone vs placebo | 0.489 | 0.348 | -1.641 | 1.813 | 2.131 | 1.879 | 0.257 |
| Lurasidone vs placebo | 0.967 | 0.286 | 0.983 | 1.059 | -0.016 | 1.138 | 0.989 |
| Olanzapine vs Placebo | 1.105 | 0.365 | 1.335 | 0.541 | -0.230 | 0.689 | 0.738 |
| Risperidone vs placebo | 1.205 | 0.516 | 0.528 | 0.538 | 0.678 | 0.784 | 0.388 |
| Ziprasidone vs placebo | 1.443 | 0.353 | 0.377 | 0.871 | 1.065 | 0.939 | 0.257 |

# Table S6. Direct and indirect comparisons for the somnolence network

|  | Direct | | Indirect | | Difference | | |
| --- | --- | --- | --- | --- | --- | --- | --- |
| Asenapine vs placebo | - 0.279 | 0.331 | 1.523 | 0.875 | -1.803 | 0.984 | 0.067 |
| Blonanserin vs placebo | 0.726 | 0.932 | 1.957 | 1.852 | -1.232 | 2.190 | 0.574 |
| Haloperidol vs placebo | 0.882 | 0.370 | 0.362 | 0.401 | 0.519 | 0.564 | 0.356 |
| Iloperidone vs placebo | 1.214 | 0.726 | -1.128 | 1.241 | 2.342 | 1.652 | 0.156 |
| Lurasidone vs placebo | 0.679 | 0.316 | 1.552 | 0.772 | -0.873 | 0.847 | 0.303 |
| Olanzapine vs placebo | 0.910 | 0.221 | 1.107 | 0.372 | -0.196 | 0.437 | 0.653 |
| Paliperidone vs placebo | 0.327 | 0.299 | -0.237 | 0.678 | 0.564 | 0.757 | 0.457 |
| Quetiapine vs placebo | 1.521 | 0.844 | 0.937 | 0.295 | 0.584 | 0.894 | 0.513 |
| Risperidone vs placebo | 0.176 | 0.599 | 0.877 | 0.301 | -0.701 | 0.672 | 0.297 |
| Ziprasidone vs placebo | 1.605 | 0.829 | 0.464 | 0.276 | 1.141 | 0.877 | 0.193 |
| Zotepine vs placebo | 1.601 | 0.525 | 1.207 | 0.695 | 0.395 | 0.871 | 0.650 |

## Supplementary Figures

# Figure S1. Risk of bias (n= 87)

#
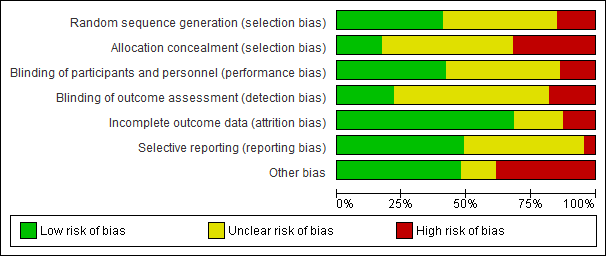


# Figure S2. Results of network rank test for insomnia

#
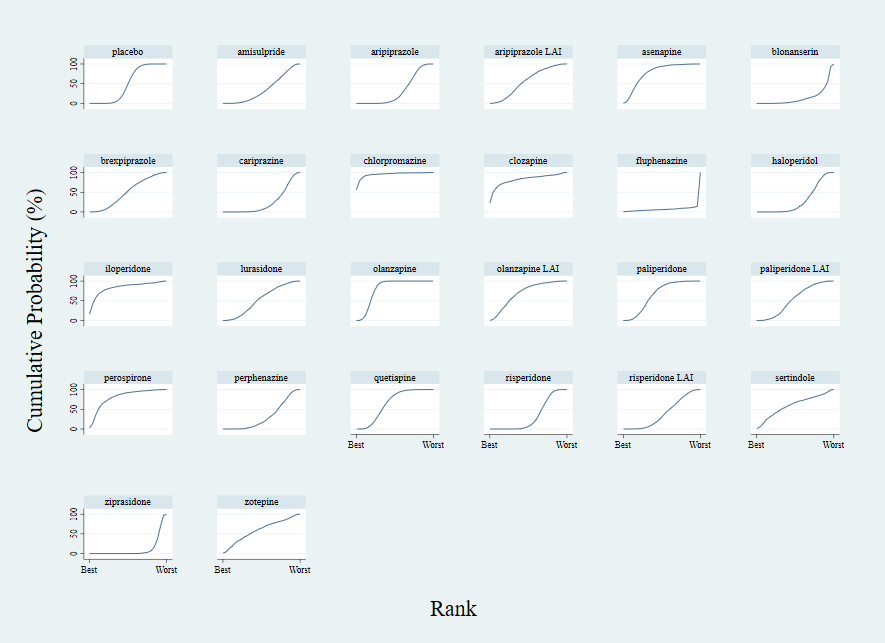


# Figure S3. Results of network rank test for somnolence

#
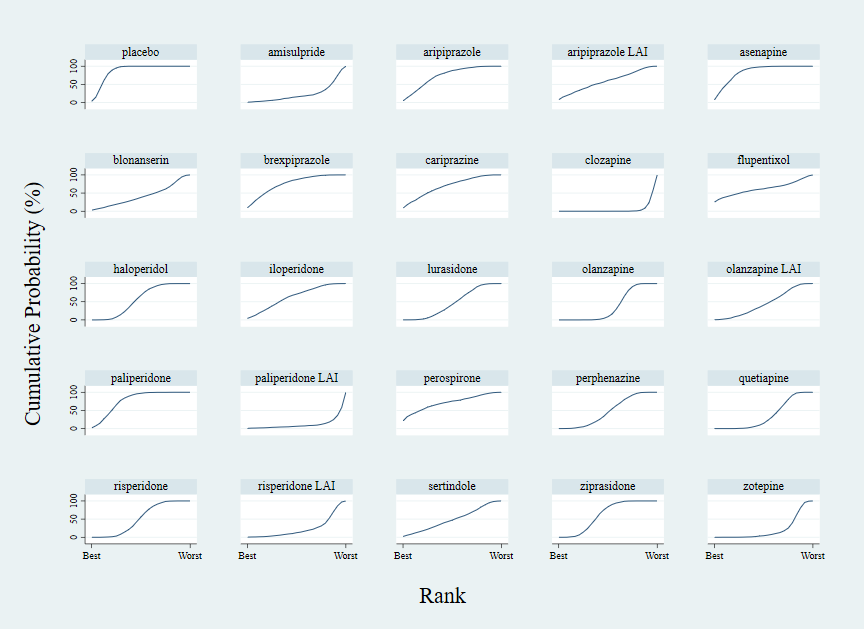


# Figure S4. Results of network rank test for sedation

#
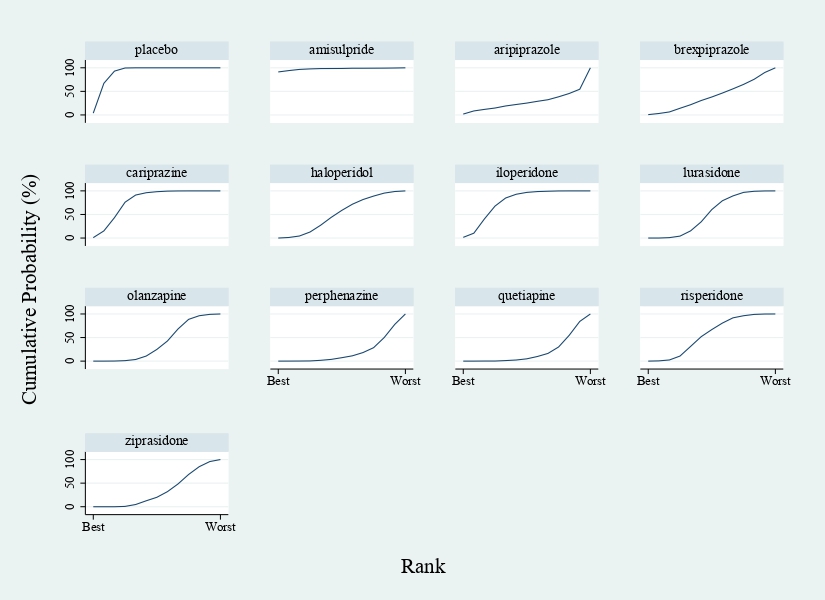

Supplement: Supplementary file 1 [file Data_Sheet_1.docx]
